# Research Advances on Anti-Cancer Natural Products

**DOI:** 10.3389/fonc.2022.866154

**Published:** 2022-05-06

**Authors:** Meng Guo, Jie Jin, Dong Zhao, Zheng Rong, Lu-Qi Cao, Ai-Hong Li, Xiao-Ying Sun, Li-Yi Jia, Yin-Di Wang, Ling Huang, Yi-Heng Li, Zhong-Jing He, Long Li, Rui-Kang Ma, Yi-Fan Lv, Ke-Ke Shao, Hui-Ling Cao

**Affiliations:** ^1^College of Pharmacy, Shaanxi University of Chinese Medicine, Xianyang, China; ^2^Xi’an Key Laboratory of Basic and Translation of Cardiovascular Metabolic Disease, Shaanxi Key Laboratory of Ischemic Cardiovascular Disease, Institute of Basic and Translational Medicine, Xi’an Medical University, Xi’an, China; ^3^Shaanxi Key Laboratory of Chinese Herb and Natural Drug Development, Medicine Research Institute, Shaanxi Pharmaceutical Holding Group Co., LTD, Xi’an, China; ^4^College of Life Sciences, Northwest University, Xi’an, China

**Keywords:** natural product, anti-cancer, core structure, derivatization, molecular mechanism

## Abstract

Malignant tumors seriously threaten people’s health and life worldwide. Natural products, with definite pharmacological effects and known chemical structures, present dual advantages of Chinese herbs and chemotherapeutic drug. Some of them exhibit favorable anti-cancer activity. Natural products were categorized into eight classes according to their chemical structures, including alkaloids, terpenoids and volatile oils, inorganic salts, phenylpropanoids, flavonoids and isoflavones, quinone, saponins and polysaccharides. The review focused on the latest advances in anti-cancer activity of representative natural products for every class. Additionally, anti-cancer molecular mechanism and derivatization of natural products were summarized in detail, which would provide new core structures and new insights for anti-cancer new drug development.

## 1 Introduction

Malignant tumors, as the second leading death cause, seriously threaten people’s health and life worldwide. It was estimated that there were approximately19.3 million new cancer cases and 10 million cancer deaths worldwide in 2020 (1). Chemotherapeutic drugs are the major therapy, which includes cytotoxic drugs, hormonal drugs, biological response regulators, monoclonal antibodies, adjuvants and others. Although they suppress tumor growth, their adverse toxic effects frequently affect patients’ health and life quality. For instance, renal or liver injury, myocardial cell contractile dysfunction, abnormal blood coagulation, serious gastrointestinal reactions and so on. New target drugs significantly promote survival rate of cancer patients, but they are susceptible to drug resistance (2).

There are about 11,146 medical plants in China and 200 ones exhibit anti-cancer activity, such as Radix Sophorae Subprostratae, Black Nightshade, Taxus mairei, etc (3, 4). Natural products are pharmacological components extracted and separated from medical plants, animals or minerals and identified chemical structures by chemical and physical techniques. On the one hand, as major active components in Chinese herbs, they exhibit definite pharmacological effects and high security based on thousands of clinical practice. On the other hand, they have known chemical structure to facilitate new drug development (5, 6). As a result, natural products present dual advantages of Chinese herbs and Chemotherapeutic drugs. Some of them exhibit favorable anti-cancer activity and would provide new core structures and new insights anti-cancer new drug development. Natural products were divided into eight categories according to their chemical structures, including alkaloids, terpenoids and volatile oils, inorganic salts, phenylpropanoids, flavonoids and isoflavones, quinone, saponins, polysaccharides (3–6). The review summarized research advances in anti-cancer activity of representative natural products for every class and focused on their anti-cancer molecular mechanism and derivatization, which would provide new core structures and new insights for anti-cancer new drug development.

## 2 Anti-Cancer Functions of Natural Products

Many natural products exhibit anti-cancer activity, which are divided into eight categories according to their chemical structures. (1) Alkaloids, such as harringtonine, camptothecin, vincristine, matrine, evodiamine and evodiamine. (2) Terpenoids and volatile oils, such as artemisinin, paclitaxel and triptolide. (3) Inorganic salts, such as As_2_O_3_. (4) Phenylpropanoids, such as podophyllotoxin. (5) Flavonoids and isoflavones, such as genistein, apigenin, and baicalein. (6) Quinones, such as tanshinone and emodin. (7) Saponins, such as ginsenoside. (8) Polysaccharides, such as lycium barbarum polysaccharide and lentinan (3–6). The review focused on research advances in anti-cancer activity of representative natural products for every category, which was expected to provide new core structures and new insights for anti-cancer new drug development.

### 2.1 Alkaloids

Alkaloids refer to a class of natural products with N-atom in the chemical structure, such as harringtonine, camptothecin, vincristine, matrine, evodiamine and rutaecarpine. The alkaloids are divided into eight categories according to their structures, as shown in **Table 1** (6–8).

**Table 1:** Classification of anti-tumor alkaloids and representative natural products.

Category		Representative natural products
Ornithine alkaloids	Pyrrolidines	Orcosine
	Scopolanes	Scopolamine
	Pyrrolizidine	Senecioine
Lysine alkaloids	Piperidines	Piperine
	Quinolizidine	Matrine
	Indolizidines	Monophylline
Phenylalanine and tyrosine alkaloids	Amphetamines	Ephedrine
	Isoquinolines	Berberine
	Benzyl phenethylamines	Lycoline
Tryptophan alkaloids	Simple indoles	Indigoside
	Dimeric indoles	Camptothecin, vincristine
	Other indoles	Evodiamine
Anthranilic acid alkaloids	Quinolines	Dicerine
	Acridones	Caprinine
Histidine alkaloids		Pilocarpine
Terpenoid alkaloids		Gentioline, Aconitine
Steroidal alkaloids		Solanine

#### 2.1.1 Harringtonine

**Figure 1:**
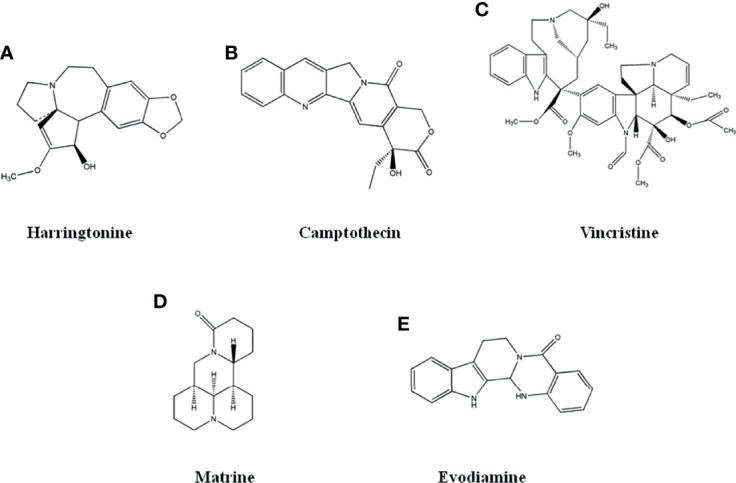
The structure of Alkaloid compounds. **(A)** Harringtonine. **(B)** Camptothecin. **(C)** Vincristine. **(D)** Matrine. **(E)** Evodiamine.

Cephalotaxus herbs has been used for anti-cancer clinical practice in ancient China. Harringtonine (**Figure 1A**), an alkaloid monomer, was extracted from Torreya Grandis in 1963. In 1973, it was demonstrated that homoharringtonine had an apparent inhibition on mouse lymphocytic leukemia cell line P388 and leukemia cell line L1210. Homoharringtonine has been used clinically since 1974 and has shown favorable curative effects on acute and chronic myeloid leukemia, non-lymphocytic leukemia, acute promyelocytic leukemia, acute monocytic leukemia, malignant lymphoma, and others. It was added to the Chinese Pharmacopoeia in 1990. In 2012, Harringtonine was approved by FDA (American Food and Drug Administration) for the treatment of acute myeloid leukemia in 2012 (9, 10).

It was indicated that Harringtonine exhibited favorable anti-cancer activity (11–14). It inhibited cervical cancer cell line HeLa proliferation and promoted cell apoptosis by down-regulating the expression of CenpB (centromeric protein) to prevent cell cycle progression from G2 to G1 phase (11). Harringtonine induced L1210 cell cycle arrest in G1 phase and induced HL60 cell cycle arrest in G2/M phase by down-regulating the expression of cycle related proteins such as cyclinB1 and Cdc2 (12). It promoted acute promyelocytic leukemia cell line NB4 apoptosis, which was related to the down-regulation of Mcl-1 expression in NB4 cells, had nothing with apoptosis proteins Bcl-2 or Bax (13).

Cancer cell produced drug resistance attributed to overexpression of MDR1 (multidrug resistance gene1). Harringtonine reversed drug resistance of doxorubicin-resistant human gastric cancer cell line SGC-7901/ADM [relative reversal rate, (72.44±2.92)%] by inhibiting MDR1 expression to promote cell apoptosis (14).

#### 2.1.2 Camptothecin

Camptothecin (**Figure 1B**), a pentacyclic dipoly-indole alkaloid, was first extracted from Camptothecus in 1966. It was indicated that Camptothecin had a broad-spectrum anti-cancer activity and was used for the treatment of gastric cancer, rectal cancer, and leukemia. Irinotecan, a camptothecin derivative, was launched in Japan in 1994 and was approved by US FDA two years later. It has remarkable curative effects on advanced colorectal cancer. Currently, irinotecan is the first-line clinical therapy for colorectal cancer, lung cancer, breast cancer, etc (15–20).

It was indicated that combining Camptothecin or its derivatives with other drugs established favorable anti-tumor activity, such as inhibiting the energy metabolism of tumor cells, inducing cell cycle arrest, and promoting apoptosis. The mechanism was related to the up-regulation of phosphorylation of associated proteins, such as Akt (protein kinase B), p38MAPK (mitogen-activated protein kinases), and ERK (extracellular regulated protein kinases). It is also related to the activation of the caspase-dependent pathway, downregulating the expression of anti-apoptotic protein Bcl-2, and upregulating the expression of pro-apoptotic protein Bax and cleaved caspase-3 (15–20). Combining Bufalin and Hydroxycamptothecin reduced the cell cycle arrest in G2/M and S phases in human prostate cancer cell line DU145, increasing the expression of caspase-3 and caspase-9 and inhibiting cell proliferation (15). The inhibitory effects of Hydroxycamptothecin on HeLa cells transfected with P53 gene was significantly enhanced, and the P53 gene could promote the pro-apoptosis effects of Hydroxycamptothecin on HeLa cells (16). The combination of Hydroxycamptothecin (0.625 µmol/L) and Celecoxib (30 mg/L) could promote cell apoptosis of human hepatoma cell line SMMC-7721 by down-regulating the expression of Bcl-2 and COX-2, up-regulating the expression of Bax (17). Hydroxycamptothecin combined with 2-DG (2-deoxy-D-glucose, 5 mmol/L) could inhibit the energy metabolism and promote cell apoptosis of human breast cancer cell lines MDA-MB-231 and MCF-7 by up-regulating the expression of pro-apoptotic protein caspase-3 (18). The combination of Camptothecin and Chonglou Saponin II could promote cell apoptosis of cell lines H460 and H446 by up-regulating the phosphorylation of Akt, P38MAPK, ERK to down-regulate the expression of anti-apoptotic protein Bcl-2 (19). The treatment with different concentration Camptothecin could affect cell morphology and increase the cell early apoptosis rate of human prostate cancer cell line PC-3 (IC_50_, 23.25 µM) by affecting the expressions of Bax, cleaved caspase-3 and Bcl-2 (20).

#### 2.1.3 Vincristine

Vincristine (**Figure 1C**), a dimeric indole alkaloids extracted from Catharanthus roseus, was approved by US FDA in 1963 as a microtubule inhibitor. It is mainly used for the treatment of acute lymphoblastic leukemia and Hodgkin lymphoma. It is also used in the treatment of germ cell tumors, small cell lung cancer and breast cancer (21–26).

The combination of Vincristine or its derivatives with other drugs showed favorable anti-tumor activities. The combination of Vincristine and pantoprazole could reverse drug resistance of KB/VCR-resistant cells by inhibiting the drug efflux of P-gp, inhibit cell invasion and metastasis by down-regulating the expression of MMP2 and MMP9, induce cell cycle arrest in G2/M phase by up-regulating P21expression to inhibit the phosphorylation of CyclinB1 and CDC2, promote cell apoptosis by down-regulating Bcl-2 and Bcl-xL and up-regulating PARP (poly ADP-ribose polymerase), caspase-3 and Bax (21). Vincristine combined with DDP (cisplatin) or quercetin could increase the inhibitory rate on human colorectal adenocarcinoma vincristine-resistant cell line HCT-8/V from 24.3% to 55.3% and 45.4% (22). They reversed the drug resistance of HCT-8/V cells by down-regulating the expression of P-gp to inhibit its drug efflux function and inhibited the proliferation of HCT-8/V cells and promoted apoptosis by regulating autophagy (23). Vincristine combined with Celecoxib could significantly inhibit the proliferation and migration (24) and promote cell apoptosis of oral cancer drug-resistant cell KB/VCR by down-regulating the expression of the anti-apoptotic protein Bcl-2 and up-regulating the expression of the pro-apoptotic protein Bax (25, 26).

#### 2.1.4 Matrine

Matrine (**Figure 1D**), a quinolizidine alkaloids, was extracted from the dried root of Sophora flavescens, a leguminous plant. Matrine has many pharmacological activities, such as anti-tumor, anti-virus, immune regulation, etc. It is used as an auxiliary anti-tumor drug in clinics (27).

Both Matrine or Oxymatrine presented a favorable anti-tumor activity (28–32). Phosphorylation of Histone H2AX (γH2AX) at ser139 site was essential for tumor cell proliferation and migration. Matrine or Oxymatrine could inhibit the proliferation and migration of human cervical cancer cell lines SiHa and c33a (28), which was related to the activation of p38 signaling pathway, up-regulation of the phosphorylation level of H2AX (28). Promoting EMT (epithelial-mesenchymal transition) can promote the invasion and migration of tumor cells. Oxymatrine could inhibit the invasion and metastasis of human pancreatic cancer cell line Panc-1 by inhibiting EMT, which was related to up-regulation of E-cadherin, down-regulation of vimentin, mucin 1, Snail (full-length protein) and Gli2 (core transcription factor) in Twist and hedgehog signaling pathways (29). Matrine or Oxymatrine induced cell cycle arrest, which was related to up-regulation of the expression of p21 gene, and inhibition of cell DNA synthesis and proliferation. Mucin 1 is highly expressed in tumor cells and a marker protein for normal cells to turn to tumor cells. Matrine combined with radiotherapy could down-regulate the mRNA and protein expression of mucin 1 in HepG2 cells, and significantly increase apoptosis rate of HepG2 cells (30). Matrine could induce cell cycle arrest at the G1 phase in human endometrial carcinoma cell line Ishikawa (IC_50_, 20.66 μg/mL) (31). P21 gene is the upstream gene of CDKs (cell cycle-dependent kinases), which is closely related to the cell division. Oxymatrine could up-regulate the expression of the p21 gene in human gastric cancer cell line SGC-7901 to inhibit cell DNA synthesis and proliferation, and could induce SGC-7901 cell cycle arrest in G0/G1 phase (32).

#### 2.1.5 Evodiamine and Rutaecarpine

Evodiamine and rutaecarpine (**Figure 1E**), indole alkaloids extracted from Evodia rutaecarpa, are used in clinical treatment for anti-cancer, gastric ulcer and oral ulcer. Evodiamine had inhibitory activity on various tumor cells with few toxicity (33–38).

Cyclin cdc25c is a key molecule in cell cycle regulation, promoting cells to enter the M phase. Evodiamine could down-regulate the expression of cyclin cdc25c and up-regulating the expression of p53 to induce human gastric cancer cell line BGC-823 cell cycle arrest in G2/M phase. It could promote cell apoptosis by up-regulating the expression of apoptosis-related proteins cleaved caspase-3, 8, 9 and cleaved PARP-1 (33). Evodiamine could inhibit cell proliferation, induce cell cycle arrest in the G2/M phase and promote cell apoptosis of SW1990 cell by up-regulating the expression of active caspase-3 and 8 (34). Evodiamine could promote cell apoptosis of human osteosarcoma cell line HOS by up-regulating the expression of caspase-3 and down-regulating the expression of Bcl-2 (35). Evodiamine could exhibited better inhibitory activity on the proliferation of human lung adenocarcinoma cell line A549 than cisplatin at 24 h and reached the best effects at 72 h (36). Rutaecarpine exhibited the best inhibitory effects on hepatoma cell line H22 cells at 24 h (IC_50_, 24.81 μg/ml), on sarcoma cell line S180 cells at 48 h (IC_50_, 19.35 μg/ml), on HepG2 at 72 h (IC_50_,14.52 μg/ml) (37). Rutaecarpine could inhibit the proliferation of S180 cells and H22 cells *in vivo*, and it induced thymus and spleen injury of nude mice was less than that of cyclophosphamide (38).

### 2.2 Terpenoids and Volatile Oils

Terpenoids, in the form of volatile oil, were synthesized by methylpentanedioic acid pathway, including monoterpenes and sesquiterpenes, such as artemisinin, paclitaxel and triptolide (6–8).

#### 2.2.1 Artemisinin

**Figure 2:**
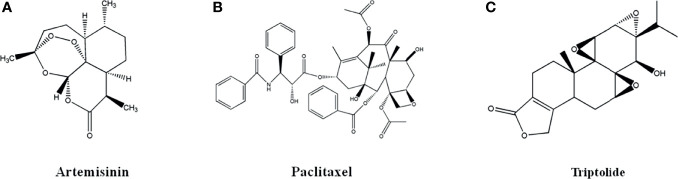
The structure of Terpenoids and Volatile oils. **(A)** Artemisinin. **(B)** Paclitaxel. **(C)** Triptolide.

Artemisinin (**Figure 2A**), a sesquiterpene lactone with a peroxy group, was extracted from the leaves of Artemisia annua L. by Chinese scholars in 1971. Artemisinin and its derivatives are well-known anti-malarial drugs and have anti-tumor activity. The artemisinin discovery is a successful example of new drug development from natural products. Youyou Tu, a Chinese pharmacologist, won the 2015 Nobel Prize in Physiology or Medicine for artemisinin discovery.

Artemisinin and Dihydroartemisinin exhibited favorable anti-tumor activity in inhibiting proliferation, migration, and invasion of tumor cells, which was attributed to up-regulating the expression of RECK (reversion inducing cysteine rich protein with Lazal motif) and down-regulating the expression of MMP-2 and N-Cadherin (39). Artemisinin inhibited the proliferation and promote apoptosis of hepatocellular carcinoma cell lines Huh7 and SMMC-7721 by inhibiting the phosphorylation of Akt and S6 in mTOR signaling pathway of Huh7 cells and the phosphorylation of C-myc and S6 in mTOR signaling pathway of SMMC-7721 cells (40). Artemisinin induced cell cycle arrest in G/2M phase in HepG2 cells (41) or induced cell cycle arrest in G0/G1 phase in human breast cancer cell line MT40 (42) by regulating the mitochondrial pathway to inhibit cell proliferation and promote apoptosis (41).

Dihydroartemisinin, an artemisinin derivative, could inhibit tumor cell proliferation, migration, and invasion and promote apoptosis (43–46). Dihydroartemisinin could inhibit the proliferation and promote apoptosis of human pancreatic cancer cell line PANC-1, which was related to the mitochondrial pathway (43). Dihydroartemisinin could inhibit the proliferation of cutaneous T-cell lymphoma cells and promote cell apoptosis (44). Dihydroartemisinin also inhibited activity on ovarian cancer cells (45) and liver cancer cells SMMC-7721 (46).

#### 2.2.2 Paclitaxel

Paclitaxel (**Figure 2B**), a diterpenoid monomer from the bark of Taxus mairei, is mainly used in the first-line clinical treatment of ovarian cancer and non-small cell lung cancer, as well as the follow-up treatment of breast cancer. It was approved by US FDA in 1992 and listed in China in 1995. Docetaxel, a drug with Paclitaxel as major ingredient, was approved by US FDA in 2004 for the treatment of breast cancer, ovarian cancer and non-small cell lung cancer. Paclitaxel exerted anti-tumor activity by inhibiting tubulin depolymerization to inhibit cell mitosis to inhibit proliferation and promote cell apoptosis (47, 48).

Paclitaxel showed favorable inhibitory activity on a variety of tumor cells (47, 48). Combined with various drugs, paclitaxel could inhibit the proliferation and migration of tumor cells through promoting the secretion of immune factors, regulating the expression of EMT-related proteins, down-regulating the expression of N-Cadherin, β-Catenin and Vimentin, and up-regulating the expression of epithelial marker proteins E-Cadherin, Claudin-1 and ZO-1 (47, 48). Paclitaxel combined with luteolin could inhibit the proliferation and migration, promote apoptosis of human esophageal cancer cell lines EC109 and TE-1, which was attributed to expression changes of EMT-related proteins, down-regulation of N-Cadherin, β-Catenin and Vimentin, and up-regulation of E-Cadherin, Claudin-1 and ZO-1 (47). The curative effects *in vivo* of paclitaxel combined with PD1 antibody was better than that of single drug therapy in xenograft nude mice model with D2F2 breast cancer (48).

#### 2.2.3 Triptolide

Triptolide (**Figure 2C**), epoxy diterpene lactones from the root bark of Tripterygium wilfordii, had effects of anti-tumor, anti-rheumatoid and anti-oxidation (49–52). Triptolide had inhibitory activity on human colorectal cancer, endometrial cancer, breast cancer, A549/Taxol lung cancer, thyroid cancer, mouse lymphoma cells, which was attributed to the expression of p53 gene, nucleus transcription factor and tumor necrosis factor, heat shock proteins, and estrogen receptor, the regulation of PI3K-Akt-mTOR, MAPK, Wnt-β-catenin signal pathways (49). Triptolide could promote apoptosis of human colon cancer cell line HCT116 cells by inhibiting Akt phosphorylation to promote autophagy (50). Triptolide could induce cell cycle arrest in G2/M phase of human endometrial cancer cell line Ishikawa, which was closely associated with the down-regulation of Akt and mTOR protein phosphorylation and mRNA in PI3K-Akt-mTOR pathway (51). Triptolide could significantly promote cell apoptosis and inhibit cell proliferation of human ovarian cancer cell line SKOV3 by increasing ROS and up-regulating the expression of cleaved caspase-9 and cleaved caspase-3, and down-regulating the expression of bcl-2 (52).

### 2.3 Inorganic Salt

Arsenic trioxide (As_2_O_3_), inorganic monomer, is highly toxic. As_2_O_3_ was another successful example for new drug development from natural products. Chen Zhu’s team treated acute promyelocytic leukemia with all-trans retinoic acid and As_2_O_3_ and the 5-year disease-free survival rate jumped to more than 90% to reach the cure level. Dr. Chen Zhu was awarded the Ernest Butler Award of the American Society of Hematology in 2015 (8).

As_2_O_3_ could inhibit cell proliferation and promote cell apoptosis in hepatoma stem cell MHCC97H by down-regulating Bcl-2 expression (53). As_2_O_3_ could promote the apoptosis of mouse melanoma cell line B16 by up-regulating the expression of p53 and Bax, down-regulating the expression of Bcl-2 (54). APC (adenomatous polyposis coli) is a tumor suppressor gene associated with colon adenomatous polyps, colon and rectal cancer and other diseases. As_2_O_3_ (2-8 μmol/L) could up-regulate the mRNA and protein expression of APC gene, therefore, effectively inhibit the proliferation of T24 cells and promote apoptosis (55). FOXO1 (forkhead boxO1) gene is a transcription factor regulating cell growth and a tumor suppressor gene. As_2_O_3_ could up-regulate the expression of FOXO1 gene mRNA and protein, thus, inhibit cell proliferation, migration and invasion, and promote cell apoptosis of human breast cancer cell line MCF-7 (56). As_2_O_3_ could inhibit cell proliferation and promote cell apoptosis of human uterine sarcoma cells by down-regulating the level of ERK phosphorylation, up-regulating the expression of caspase-3 (57).

### 2.4 Phenylpropanoids

Phenylpropanoid compounds, whose primary core structure is C6-C3 connected by a benzene ring and a 3-chain carbon. The representative compounds of Lignans are Podophyllotoxin, Isotaxol, Schisandrin, Magnolol and Phillyrin (58–60).

**Figure 3:**
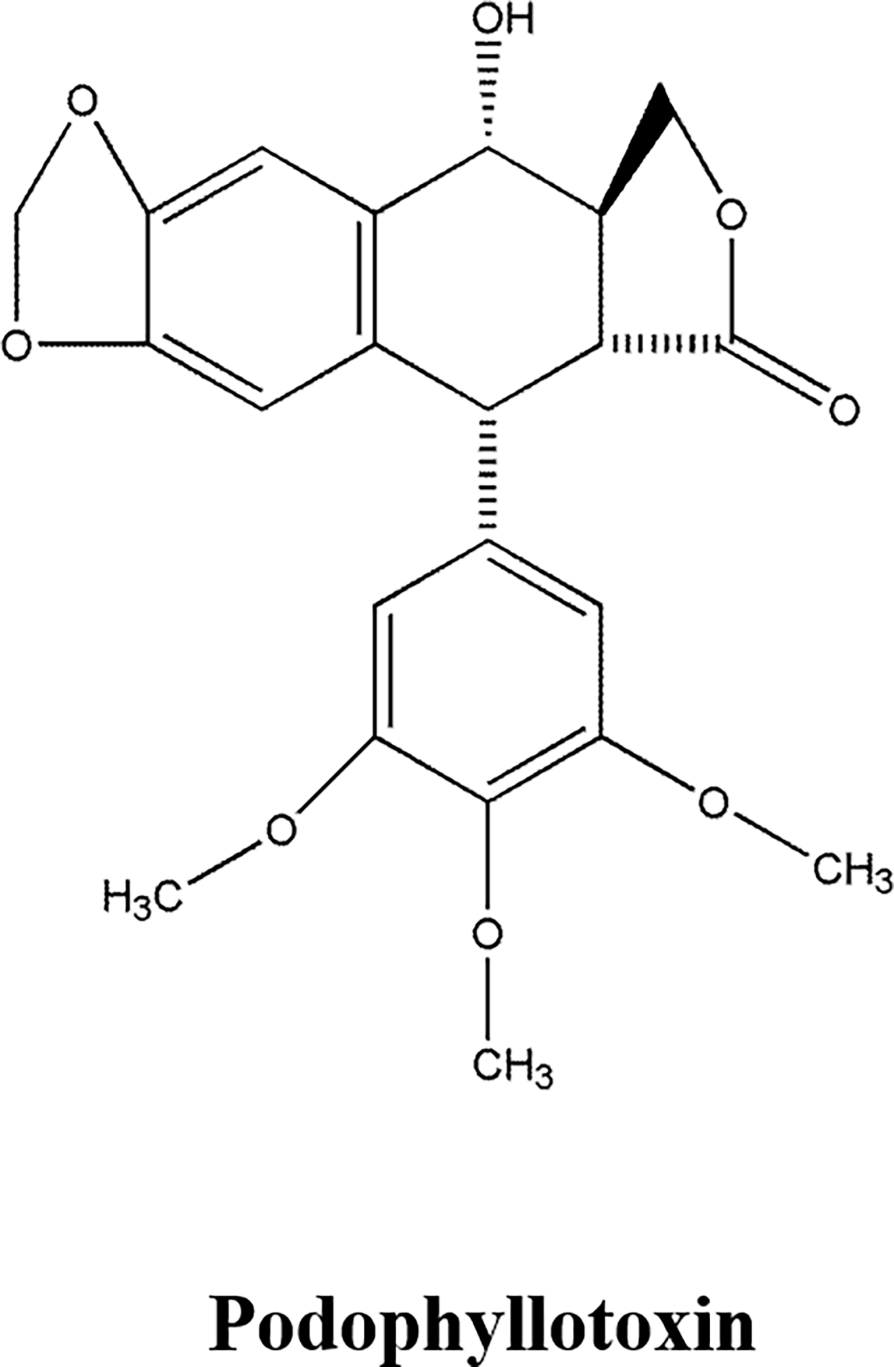
The structure of Phenylpropanoid compound. Podophyllotoxin.

Podophyllotoxin (**Figure 3**), a lignin monomer extracted from the rhizome of Common Dysosma Rhizome, had anti-tumor effects (58–60). Podophyllotoxin could inhibit tumor cell proliferation and promote apoptosis, which was attributed to inhibiting tubulin polymerization to prevent the formation of a mitotic spindle, therefore, affecting cell division, and inducing cell arrest in metaphase (58). Podophyllotoxin, Picropodophyllotoxin and 4-demethylpodophyllotoxin could inhibit the cell proliferation of human hepatoma cell lines QSG7701 and SMMU7721 and HeLa cells. Among them, Podophyllotoxin performed the best activity. Podophyllotoxin, with broad-spectrum of anti-tumor activity, had favorable inhibitory activity on human leukemia cell line Jurkat, pleomorphic glioma cell line T98G and Glioma cell line SH-SY5Y. Podophyllotoxin showed excellent inhibitory activity on HeLa cells, promoted the formation of apoptotic bodies in the nucleus, and apoptosis occurred in 48 h, and induced cell cycle arrest in G2/M phase, which was attributed to down-regulation of calreticulin and Bcl-2, up-regulation of nucleolar phosphate protein and Bax to induce cell cycle arrest and promote apoptosis (59). Deoxypodophyllotoxin could inhibit the proliferation of human ovarian cancer cell line SKOV3, which was attributed to the down-regulation of ERK1/2 protein expression (60).

### 2.5 Flavonoids and Isoflavones

Flavonoids and isoflavones, 2-phenylchromone as the core structure, include a variety of structural types, such as Flavonoids, Dihydroflavonoids, Isoflavones, Dihydroisoflavones, Chalcone, Hesperidone and so on. The representative monomers have Genistein and Apigenin (61–67).

#### 2.5.1 Genistein

**Figure 4:**
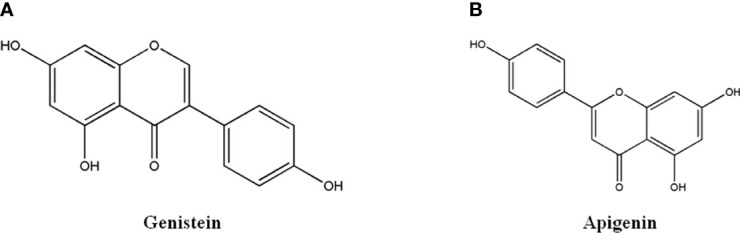
The structure of Flavonoids. **(A)** Genistein. **(B)** Apigenin.

Genistein (**Figure 4A**), a flavonoid from the rhizome of Leguminosae Genistin. It has anti-tumor, anti-bacterial, anti-oxidant, hypolipidemic and estrogen-like effects. It could inhibit angiogenesis, induce tumor cell programmed death, therefore, exhibit favorable anti-tumor activity (61–63). Genistein could reverse drug resistance of 5-fluorouracil-resistant hepatoma cell line Bel-7402/5Fu by up-regulating the expression of Akt and mTOR in PI3K/Akt/mTOR pathway, down-regulating the expression autophagy-related genes of Beclin and LC3 to promote cell autophagy (61). Genistein could inhibit the proliferation of human colon cancer cell line HCT116, which was related to the up-regulation of the ratio of LC3 II/I, the formation of autophagosome to promote cell autophagy (62). Genistein derivative WH-20 could inhibit the proliferation, migration and invasion of human breast cancer cell line MCF-7 and induce cell cycle arrest in G1 phase, which was attributed to down-regulated expression of related proteins in Notch pathway, such as Notch1, Jagged1, NF- κB, p65 and IκBa. Additionally, it inhibited the proliferation, migration and induced cell cycle arrest of MCF-7 cells by binding to Er β receptor, down-regulating the expression of MMP9, VEGF and CyclinD1 (63).

#### 2.5.2 Apigenin

Apigenin (**Figure 4B**), Flavonoid monomer widely found in plants, has anti-tumor, anti-bacterial, anti-viral, and cardio-cerebrovascular protective effects (64–67). Apigenin could significantly inhibit the proliferation and invasion of human esophageal cancer cell line Eca-109 (65) and human lung cancer cell line PC9GR (66) by up-regulating the expression of E-cadherin, and down-regulating the transcription factor Snail, MMP-9 and VEGF. Apigenin could inhibit the growth of 3D tumor sphere of human hepatocellular carcinoma cell line MHCC97H by up-regulating the protein expression of SHP-1, down-regulating the phosphorylation level of STAT3 protein (67).

### 2.6 Quinonoid

The quinonoid monomers had favorable anti-cancer activity, such as Tanshinone and Emodin (68–76).

#### 2.6.1 Tanshinone

Tanshinone, phenanthrenequinones derived from Salvia Miltiorrhiza, included Tanshinone IIA, Isocryptotanshinone, and Dihydrotanshinone, which exhibited anti-cancer, anti-bacterial, anti-inflammatory activities (68–73).

Tanshinone monomers could inhibit the proliferation of human colorectal cancer and gastric cancer cells, induce cell cycle arrest, and promote cell apoptosis, which was related to downregulating the expression level of HIF-1 (hypoxia inducible factor-1), VEGFR, bFGF (68), inhibiting tumor angiogenesis, suppressing the expression level of transcription related protein, down-regulating anti-apoptotic protein Bcl-2, while upregulate the pro-apoptotic proteins p53, Bax, cleaved caspase-3, and p21. They could reverse the doxorubicin drug resistance in gastric cancer cells by downregulating the expression of MRP1 and p62, while upregulating the autophagy-related gene LC3B-II to promote cell autophagy (69).

Tanshinone IIA inhibited the proliferation of HCT-116 cells by downregulating the expression of HIF-1α, VEGFR, and bFGF to inhibit angiogenesis (68). Combination of Tanshinone IIA and As_2_O_3_ synergistically inhibited the proliferation of SW620 cells by downregulating the expression of MMP9, VEGF, CD44v6, and upregulating the nm23-H1 (69). Phosphorylation of STAT3 promoted the progression of gastric cancer. Tanshinone IIA inhibited the proliferation and promoted apoptosis of human gastric cancer cell lines SNU-638, MKN1, and AGS cells, which was attributed to inhibiting the phosphorylation of STAT3 to downregulate Bcl-2, and upregulate Bax, and cleaved caspase-3 (70). Tanshinone IIA reversed the doxorubicin resistance in SNU-719R and SNU-610R cells by decreasing the expression of MRP1, inducing cell cycle arrest in G2/M phase, downregulating the expression of Bcl-2, while upregulating the level of p53 and Bax to promote cancer cell apoptosis, upregulating the autophagy-related protein LC3B-II and downregulate p62 to facilitate cell autophagy (71). Dihydrotanshinone inhibited the cell proliferation of SW480 by downregulating the β-catenin downstream protein c-Myc to inhibit the Wnt/β-catenin signaling pathway (72). Isocryptotanshinone could inhibit BGC-823 cells and SGC-7901 cells, induce cell cycle arrest in G0/G1 phase and promote cell apoptosis by upregulating p53 and p21, while downregulating Cyclin D1 and Bcl-2 (73).

#### 2.6.2 Emodin

**Figure 5:**
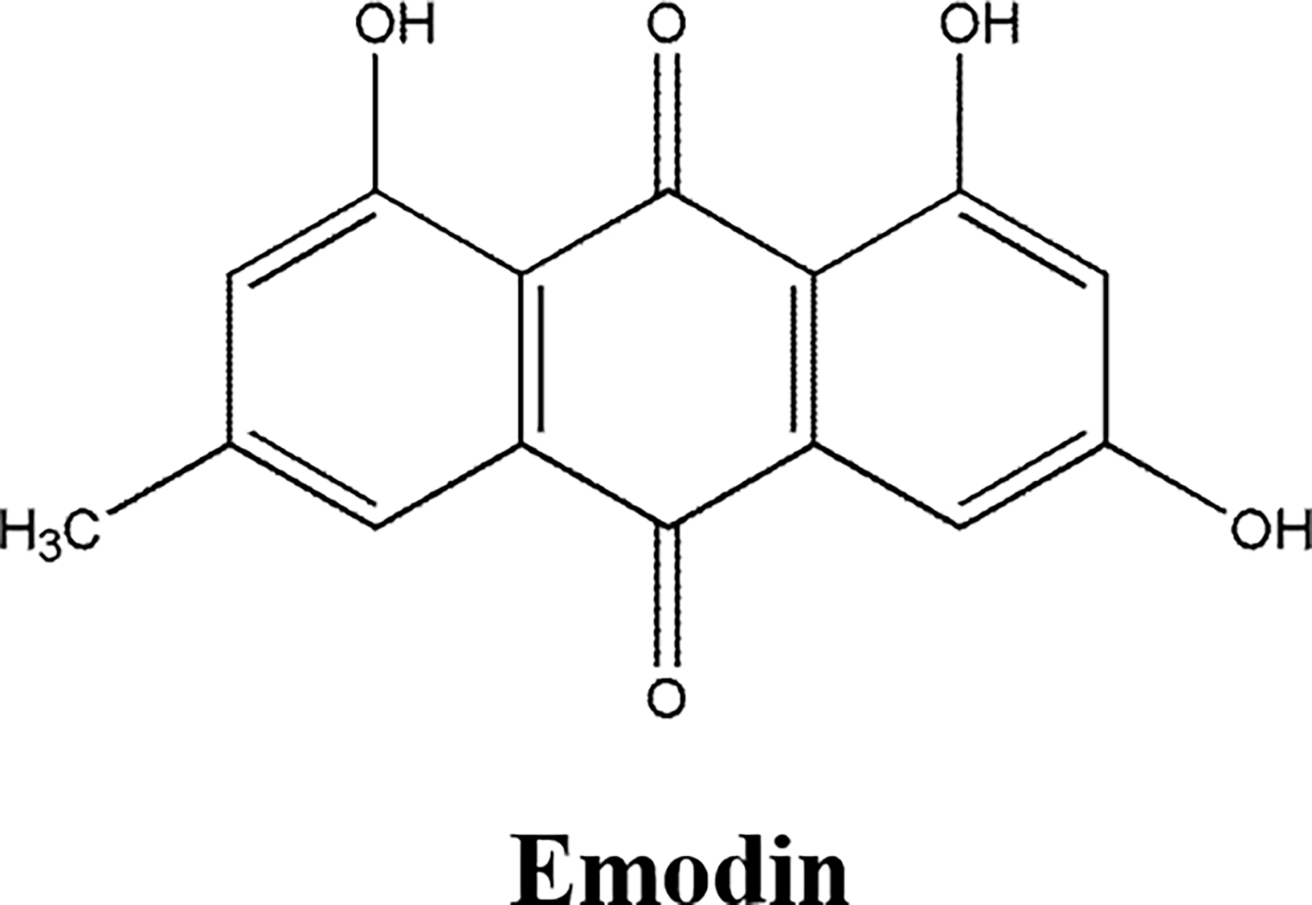
The structure of Quinone compound. Emodin.

Emodin (**Figure 5**), an anthraquinone from Rhubarb, had anti-cancer, diarrhea, anti-bacterial, anti-spasmodic, cough relief, and diuretic activity (74–76). Emodin inhibited the proliferation, migration, and invasion, suppressed the cellular glycolysis and promoted apoptosis of endometrial cancer cell line HEC-1-B and gastric cancer cell lines BGC-823 and AGS, inhibited the xenograft growth of human colon cell line CT26 in nude mice model, which was attributed to downregulating the expression of CD44 and carbonic anhydrase IX protein (74), peroxidase Prx V, increasing intracellular ROS (75), downregulating anti-apoptosis proteins Bcl-2 and Pro-caspase 3, upregulating pro-apoptosis proteins Caspase3, Bax and HIF-α (76).

### 2.7 Saponin

Saponin, foaming and hemolysis properties, included triterpenoid saponin and steroid saponin. The former included Ginesnoside and Pachymic acid, the later included Diosgenin and Polyphyllin (77–83).

Ginsenoside, derived from Panax ginseng, comprised of a wide range of saponins with anti-cancer effects such as Rh1, Rh2, Rh3, Rg3, and Rg5. Ginsenoside could reverse multidrug resistance cancer cells by regulating MDR expression (77–80). Ma et al. (77) established a multidrug resistance biliary cancer cell line QBC939/ADM using cyclophosphamide, mitomycin, and 5-fluorouracil. Ginsenoside Rg3 reversed drug resistance to inhibit cell proliferation by upregulating the expression of MDR. Wan et al. (78) established a cisplatin resistant SGC7901/DDP cell line and found that Ginsenoside Rg3 could sensitize drug resistant cells to cisplatin by downregulating the mRNA and protein level of PD-L1. Hu et al. (79) constructed a doxorubicin-resistant SGC7901/ADR cell line and found that Ginsenoside Rh2 could sensitize the cells to doxorubicin by downregulating the expression of P-glycoprotein and Bcl-2, resulting in G2/M phase arrest and apoptosis. Ginsenoside Rg3 reversed the drug resistance of Lovo/5-Fu cells by upregulating the expression of NM23 and caspase-8 without affecting the drug resistance-related protein LRP (80)..

Ginesnoside inhibited the proliferation, migration, and invasion of breast cancer, colorectal cancer, lung cancer, ovarian cancer, and glioblastoma cells by inhibiting VEGF, angiogenesis, and downregulating MMP-9 (81–83). Ginsenoside Rh1 inhibited cell migration and invasion of SW480 cells by downregulating the expression of MMP-9 (82). Ginsenoside Rh3 promoted cell apoptosis in ovarian cancer cell line SKOV-3 cells (83).

### 2.8 Polysaccharides

Polysaccharides, widely distributed in plants, animals, and microorganisms, exhibited anti-cancer activity. On the one hand, they directly inhibited cell proliferation and migration, induce cell apoptosis of cancer cells. On the other hand, they were used for anti-cancer adjuvants by activating body immune cells and complement and increasing cytokine secretion to improve the immune function (84–88).

#### 2.8.1 Lycium Barbarum Polysaccharide

Lycium barbarum polysaccharide, from medicinal and edible homologous herb wolfberry, presented anti-cancer activity. They could inhibit the proliferation, induce cell cycle arrest and promote cell apoptosis of human breast cancer cells MCF-7 (84), bladder cancer DU145 cells (85), liver cancer HepG2 cells (86), and tongue squamous carcinoma CAL-27 cells (87), which was attributed to activating the expression of ERK in p53 signaling pathway (84) and PI3K/Akt signaling pathway (85), downregulating the expression of anti-apoptotic protein Bcl-2, upregulating the expression of pro-apoptotic proteins Bax, Caspase-3 and Caspase-9, downregulating the expression of cyclinD, cyclinE and CDK2 (86), and promoting autophagy-related protein LC3 from type I to type II to promote autophagy (87). Lycium barbarum polysaccharide combined with chemotherapeutic drugs such as DDP could improve anti-cancer effects by activating body immune cells and complement and increasing cytokine secretion to improve the immune function. Additionally, they could relieve side effects of chemotherapeutic drugs (88).

#### 2.8.2 Lentinan

Lentinan, extracted from Lentinus edodes, has anti-cancer activity and improve the immune function of the body. Clinically, it has been used as adjunct therapy for advanced lung cancer and gastric cancer treatment (89–92). Lentinan could *in vivo* inhibit the growth of murine sarcoma S-180 cells, human cervical cancer HeLa cells, breast cancer MCF-7 cells, liver cancer HepG2 cells, and squamous cell carcinoma SCC-7 cells in tumor xenograft models, which was attributed to regulation of tumor suppressor p53, caspase and Era genes (89), inhibition of PI3K/Akt/mTOR signaling pathway, and inhibition of angiogenesis (90). Lentinan could inhibit the proliferation, migration, induce cell cycle arrest and promote cell apoptosis of human glioma SHG-44 cells (91), oral squamous cell carcinoma HN4 cells (92).

## 3 Anti-Cancer Molecular Mechanism of Natural Products

The anti-cancer molecular mechanism of natural products can be attributed to the regulation of (1) inhibiting the cell proliferation, migration, and invasion, inducing cell cycle arrest and apoptosis to inhibit or kill cancer cells by regulating related cell signaling pathways; (2) inhibiting angiogenesis by regulating related signaling pathway; (3) inhibiting tumor by regulating suppressor gene, autophagy, or intracellular ROS level; and (4) reversing drug resistance in cancer cells by regulating the expression level of drug resistance associated genes or transporters.

### 3.1 Regulation of Cell Proliferation, Migration, Invasion, Cell Cycle and Apoptosis by Signaling Pathways

Natural products, such as Matrine or Oxymatrine, could inhibit the proliferation and migration of cancer cells by upregulating p38 signaling pathway and phosphorylation level of H2AX (28–32). Artemisinin inhibited the proliferation of tumor cells by inhibiting the phosphorylation of Akt and S6 in mTOR signaling pathway (40–42).

EMT is a biologic process in which the polarized epithelial cells transform to a mesenchymal cell phenotype under certain conditions, leading to enhanced capability of migration and invasion. Low expression level of TGF-β was able to crosstalk with other signaling molecules and promote EMT. Cadherin, especially E-cadherin, is actively involved in the regulation of EMT and promotes cell migration. Downregulation of E-cadherin decreased the adhesiveness of cancer cells and allowed them to migrate to distal tissues (64). Paclitaxel (47), Artemisinin (39), Dihydroartemisinin, and Apigenin (66), could induce Wnt, Notch, and P38 signaling pathways, upregulate the expression of SH2-containing protein tyrosine phosphatase 1 (SHP-1) (67), RECK, β-Catenin, Vimentin, Claudin-1, ZO-1, downregulate the level of E-cadherin, p-STAT3, mucin1, Gli2, Vimentin, Snail, and Twist, therefore, inhibiting EMT to suppress cancer cell proliferation, migration and invasion.

Natural products could induce cell cycle arrest to inhibit cancer cell growth and proliferation by regulating signaling pathways to affect the cyclin expression. Paclitaxel (47) could stabilize polymerized microtubules during mitosis, leading to cell cycle arrest. The cyclin Cdc25C is an important mediator for entering mitosis and regulates G2/M. Harringtonine (13), Vincristine (21, 22), Matrine and Oxymatrine (32) and Lycium barbarum polysaccharide (84–86), could downregulate the phosphorylation of PI3K, Akt and mTOR in PI3K/Akt/mTOR signaling pathway and the protein expression in Notch pathway, inhibit the binding to Estrogen receptor β, upregulate p53, p21, and downregulate CDK2, cyclinA, cyclinB1, cyclinD1, cyclinE, Cdc2, Cdc25C, thus leading to cell cycle arrest and inhibit cancer cell growth.

Natural products could promote cancer cell apoptosis by regulating signaling pathways to affect the expression of apoptosis related proteins. Apoptosis is a process of programmed cell death, including mitochondria pathway, death receptor pathway, and endoplasmic reticulum pathway. The apoptosis related proteins include Cyto-C, Caspase3, 7, 8, 9, 12, and anti-apoptotic genes (Bcl-2, Bcl-x), and pro-apoptotic gene Bax (93). Homoharringtonine, Camptothecin, Vincristine, Artemisinin, Dihydroartemisinin and Tanshinone could downregulate Ras-MAPK, PI3k-Akt, Wnt-β-catenin, STAT3 (72), ATAD2, and Notch1, while upregulate of P53, P21, IGFBP3, NDRG1 to activate mitochondrial mediated endogenous apoptotic caspase pathway, therefore, downregulate the level of anti-apoptotic gene Bcl-2, Bcl-xL, survivin, livin (48, 76), as well as upregulate the pro-apoptotic gene Bax, cleaved caspase-3, 8, 9, AIF, and cleaved PARP1 to promote cancer cell apoptosis (33).

### 3.2 Regulation of Angiogenesis by Related Signaling Pathways

Angiogenesis is a crucial process in the development and progression of tumors, which provides essential nutrients for tumor tissues (94). Therefore, inhibiting angiogenesis has been established as an effective strategy for cancer treatment including direct ways and indirect ways. Direct angiogenesis inhibition can be achieved by downregulation of VEGF, FGF, and TNF-α, and upregulation of angiostatin, endostatin, and IF-α. VEGF is a downstream protein in the PI3K/Akt signaling pathway. Inhibiting the levels of VEGF and MMPs can decrease the degradation of extracellular matrix, which indirectly inhibits tumor angiogenesis. Ginsenosides (81–83), Tanshinones (68) and Lentinan (95) could inhibit Akt phosphorylation and the corresponding PI3K/Akt/mTOR pathway, inhibit phosphorylation of IκBα, NF-κB-p65, upregulate the expression of SHP-1, downregulate the protein level of angiogenesis-associated HIF-1α, MMP-2, MMP-9, VEGF, bFGF and Cyc-D1 to inhibit angiogenesis and the proliferation of tumor cells.

### 3.3 Regulation of Tumor Suppressor Gene, Autophagy, or Intracellular ROS Level

Natural products such as As_2_O_3_ (53–56) and Triptolide (49) could upregulate tumor suppressor genes PTEN, p53 (54), APC (55), FOXO1 (56), and miR-145, and downregulate MMP2 and MMP9 to inhibit tumor angiogenesis, migration, and invasion. Furthermore, natural products could downregulate the expression of CDK2, cyclin A, cyclin B1, cyclin D1, and cyclin E, leading to cell cycle arrest. They also induce cancer cell apoptosis by upregulating the cytochrome C, AIF, caspase-3, caspase-9, Bax and the ratio of Bax/Bcl-2 and downregulating the expression of Bcl-2.

Vincristine (23), Genistein (61), Lycium barbarum polysaccharide (87) would upregulate the expression of Akt and mTOR in PI3K/Akt/mTOR signaling pathway (61, 85, 87), while downregulating the autophagy related Beclin1, thereby promoting the transition of LC3 I to LC3 II, and mediating the formation of autophagosome. Hence, Chinese herb monomers could facilitate cancer cell autophagy, inhibit cell growth (87).

Paclitaxel (47) and Emodin (74) could promote cancer cells apoptosis *via* increasing cellular ROS level by upregulating the expression level of JNK, MAPK4, and ASK1 (47), decreasing the level of CD44 and CAIX (74), and downregulating the expression of peroxidase PrxV.

### 3.4 Regulation of Drug Resistance Associated Genes or Transporters

MDR refers to the phenomenon that cancer cells become resistant to multiple drugs that shares similar chemical structure, leading to ineffective of the drugs and increase the therapeutic burden of patients. Therefore, it is essential to sensitize cancer cells to therapeutic drugs and reverse drug resistance (96). Overexpression of MDR1 gene may lead to the MDR phenotype of cancer cells, which allows the cells to pump out chemotherapeutic drugs using the energy derived from ATP hydrolysis. Harringtonine (14), Vincristine (21) and Tanshinone (71) could downregulate the expression of MDR1 gene as well as the protein level of P-gp (21) and MRP1 (71), thereby inhibiting the drug efflux activity and reverse MDR.

## 4 Derivatization of Natural Products

Although a wide variety of natural products exhibit anti-cancer activity, the effect is not enough to use for clinical treatment. Through structural optimization, some natural products have been advanced as effective anti-cancer drugs. For example, Homoharringtonine, a derivative of Harringtonine, was the first anti-cancer natural product in China used as a first-line treatment for acute myeloid leukemia in combination with chemotherapy. It was also used for the treatment of malignant lymphoma, choriocarcinoma, malignant mole, lung cancer, and others. Irinotecan and Topotecan, the water-soluble derivatives of Camptothecin, were used for metastatic colorectal cancer. The structural derivative of Vinblastine, Isovinblastine, was approved in France in 1991 for late-stage lung cancer treatment with low neurotoxicity compared to Vinblastine. The Paclitaxel derivative Docetaxel was the first marketed Taxane drug with 2-3-fold anti-tumor efficacy than Paclitaxel. Etoposide was the first marketed anti-tumor drug of Podophyllotoxin derivatives. Many of these structural optimized monomers are still used in the first-line treatment, which is encouraging for natural products research.

### 4.1 Harringtonine Derivatives

A wide range of Harringtonie derivatives have been developed to enhance the anti-cancer efficacy since its discovery. Homoharringtonine, a derivative of Harringtonine, was the first anti-cancer Chinese herb monomer drug in China as a first-line treatment for acute myeloid leukemia, which was used in combination with chemotherapy. It was also used to treat malignant lymphoma, choriocarcinoma, malignant mole, lung cancer, and others (97–99).

Zhong et al. (97) synthesized 10 amino acid Harringtonines, which showed inhibitory effect against promyelocytic leukemia HL-60 cells. The anti-cancer activity of compound 6 showed an 75.2% inhibitory effect, suggesting the anti-cancer effects may be dependent on the compound. Ye et al. (98) synthesized 6 new ester base compounds by linking the side chain of Paclitaxel and its enantiomer to the C3 position of Harringtonine. The MTT cell viability assay showed that compound VIIIa, VIIIb, IXa and IXc showed potential anti-proliferative effects toward oral epithelial squamous cell carcinoma KB cells, CRC HCT-8 cells, and liver cancer BEL-7402 cells. Compound VIIIa has the most significant effect against HCT-8 and BEL-7402 cells. Wu et al. (99) synthesized 17 compounds by modifying Homoharringtonine at different positions. The *in vitro* data showed that the cycloheptatrienone and lactone structure of Homoharringtonine was important for the anti-cancer effects against HCT-116, A375, A549 and Huh-7 cells. Compound 6 had the most significant effects with IC_50_ values below 10 µM in cancer cells, and 67.20 µM in normal cell L-02, suggesting a good selectivity and safety for cancer treatment.

### 4.2 Camptothecin Derivatives

Camptothecins are potential anti-cancer drugs, the derivatives of which have been approved in clinical use, including 10-hydroxycamptothecin, Belotecan, Irinotecan, Topotecan. Irinotecan and Topotecan are water-soluble derivatives of Camptothecins that are used for metastatic colorectal cancer (100). Studies reported that the modification of Camptothecin is mainly focused on the ring structures, and substitution on C-9 and C-10 positions increase the anti-cancer activity of the compounds (101).

**Figure 6:**
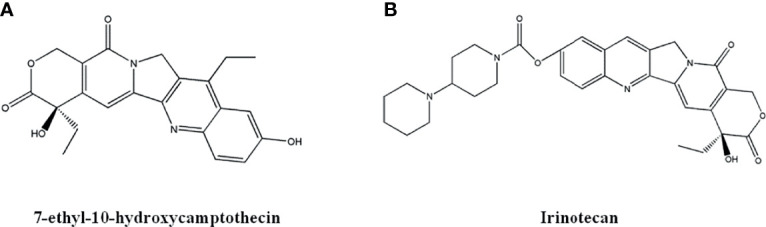
The structure of Camptothecin derivatives. **(A)** 7-ethyl-10-hydroxycamptothecin. **(B)** Irinotecan.

The derivative 7-ethyl-10-hydroxycamptothecin, also known as SN-38 (**Figure 6A**), has the strongest anti-cancer effects. Irinotecan (**Figure 6B**) is a derivative of SN-38 which is metabolized into SN-38 after administration. SN-38 showed optimal effects against metastatic colorectal cancer, small cell and non-small cell lung cancer, and breast cancer (102, 103). It is reported that the combination of SN-38 and other anti-cancer drug has no observed side effect to fetus when treating breast cancer during pregnancy (104). Guerrant et al. (105) synthesized a novel camptothecin-like histone deacetylase and topoisomerase I dual inhibitor, which demonstrated enhanced cytotoxicity against liver cancer Hela cells. Lee et al. (106) designed some novel 7-(N-substituted-methyl)-camptothecin derivatives that show significant cell killing effects towards MDA-MB-231, KB, A549, and drug resistant Kbvin cells. Wang et al. (107) synthesized a new compound with seven-membered lactone ring, which exhibited better *in vitro* anti-tumor effects than SN-38.

### 4.3 Vinblastine Derivatives

Both Vinblastine and Vincristine have significant anti-tumor activity, and the derivatives that have been approved for clinical use include Vinorelbine, Vindesine, and Vinflunine. The structural derivative of Vinblastine, Isovinblastine, was approved in France in 1991 for late-stage lung cancer treatment with low neurotoxicity compared to vinblastine. Hu et al. (108) synthesized 30 lead compounds by modifying the C-3 and C-4 position of Vinorelbine. The *in vitro* data showed that C-3 substitution has significant impact on the cytotoxicity in Hela and A549 cells. Among them, the derivatives with N25 substituent had the strongest *in vitro* anti-cancer activity, and the IC_50_ value was one third of that of the positive control. The A549 tumor xenograft model results showed that N25 substituent derivative had higher inhibitory rate than Vinorelbine and other compounds (N8, N11, N18). Li et al. (109) optimized the synthesis and separation process of 3-phenethyl ester-6’-oxyvinblastine nitrogen oxide, and the MTT assay showed promising *in vitro* anti-cancer efficacy in HepG2, Hela, MCF-7, and A549 cells. Compared to Vinorelbine, the compound had IC_50_ values lower than 10 μg/mL. Cell-penetrating peptides can covalently transport compounds into cells. Studies reported that the combination of Vinblastine with Oligoarginine had better efficacy against leukemia cell HL-60 *in vitro*, and the combination of Vinblastine and tryptophan Br-vind-(L)-Trp-Arg8 had significant inhibitory effects against leukemia cell P388.

### 4.4 Taxane Derivatives

**Figure 7:**
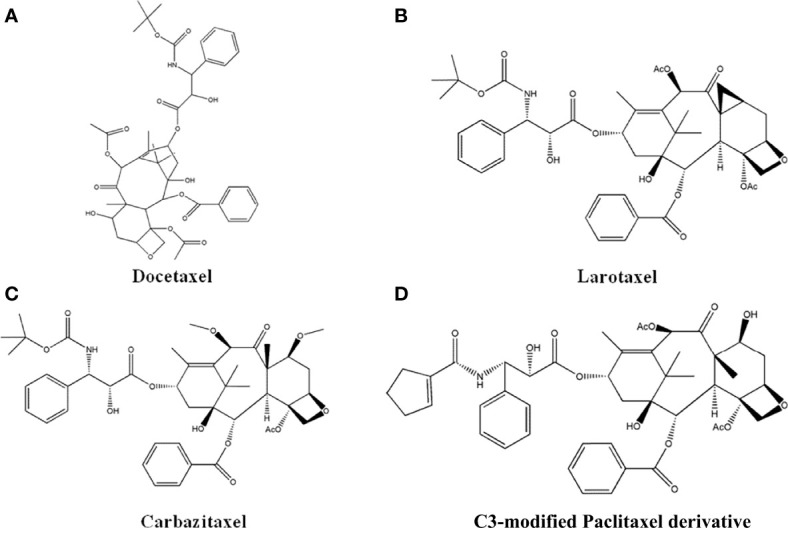
The structure of Taxol derivatives. **(A)** Docetaxel. **(B)** Larotaxel. **(C)** Carbazitaxel. **(D)** C3-modified Paclitaxel derivative.

Paclitaxel has strong anti-cancer effects, and the most successful derivative is Docetaxel, which is the first marketed semi-synthetic Paclitaxel derivative. Docetaxel (**Figure 7A**) has 2-3-fold stronger effects compared to Paclitaxel, with long retention time in cells, high bioavailability, and few side effects (110). Later, researchers have synthesized new compound by modifying the structure of Docetaxel. Che et al. (111) synthesized larotaxel (**Figure 7B**) by modifying the C-7 and C-8 position of Docetaxel, which showed promising inhibitory effects against breast cancer and pancreatic cancer. Iimura et al. (112) introduced methoxy group to the C-7 and C-10 position of Docetaxel to obtain a new compound named Carbazitaxel (**Figure 7C**). It has been approved for the treatment of hormone-resistant metastatic prostate cancer, and had favorable anti-cancer effects towards colorectal cancer, lung cancer, and cervical cancer. Roh EJ et al. (113) performed structural modification on the C-3 position of Paclitaxel, and the new compounds (**Figure 7D**) exhibited 20 times higher inhibitory effects to A549 and SK-OV3 cells than Paclitaxel.

### 4.5 Podophyllotoxin Derivatives

The structure-optimized derivatives of Podophyllotoxin showed favorable anti-tumor activity, and Etoposide was the first podophyllotoxin derivatives approved for use. Wu et al. (114) synthesized 23 derivatives by esterification and amidation of Podophyllotoxin with Ligustrazine and different amino acids. The *in vitro* experiments showed that compound P-02 had the highest inhibitory effects and induced early apoptosis in A549 and L-02 cells. Gao et al. (115) examined the anti-cancer activity of ZM-10, a derivative of Podophyllotoxin, on oral squamous cell carcinoma KB cells. The results suggested that ZM-10 could induce G2/M phase arrest and promote cell apoptosis. PCR data showed that ZM-10 downregulated the mRNA level of anti-apoptotic gene Bcl-2 and upregulated the mRNA level of pro-apoptotic gene P53, caspase-3, and Bax. Leng et al. (116) reported that QW-83 could concentration-dependently inhibit the proliferation of ovarian cancer He-La cells and induce cell apoptosis. Tian et al. (117) showed that the synthetic compound 4b, 4e, and 4f from Podophyllotoxin and indoles showed more significant cytotoxic effects on HeLa and K562 cells than Etoposide.

### 4.6 Matrine Derivatives

**Figure 8:**
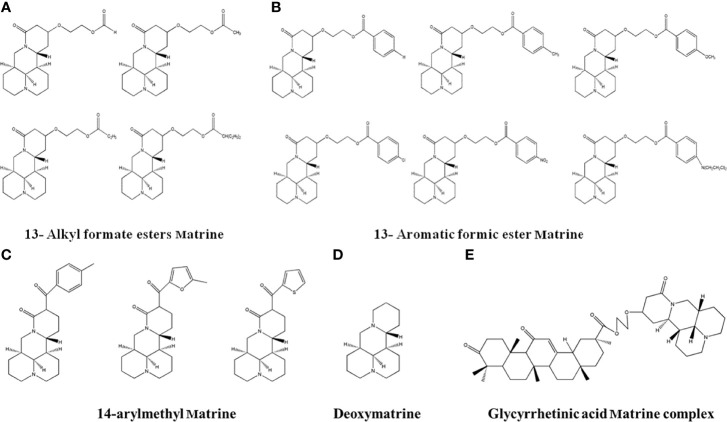
The structure of Matrine derivatives. **(A)**13-Alkylformate esters Matrine. **(B)** 13-Aromatic ester Matrine. **(C)** 14-arylmethyl Matrine. **(D)** Deoxymatrine. E. Glycyrrhetinic acid matrine complex.

Matrine is a potential anti-cancer drug. Matrine injection has been used as an adjuvant therapy in clinics since 2003. However, Matrine has a short duration of activity due to its high solubility and rapid elimination. In order to optimize the therapeutic effects, researchers performed structural medication on matrine. Wang et al. (118) obtained two types of Matrine derivatives by esterification to 13-hydroxyethyl Matrine (**Figures 8A, B**). The results showed that the derivatives parabens and 4-chlorobenzoates had more significant inhibitory effects than Matrine in HepG2 cells. Another group synthesized three 14-aroylmatrine compounds (**Figure 8C**) by introducing benzylidene derivatives to the C-14 position of matrine by Claisen-Schmidt reaction (119). The new compounds have potential anti-cancer effects to melanoma B16-F10 cells. He et al. (120) obtained Deoxymatrine (**Figure 8D**) by reducing the C-15 position of Matrine with lithium aluminum tetrahydrogen. The synthesized deoxymatrine showed concentration-dependent inhibitory effects to liver cancer HepG2 and SMMC7721 cells, and the effects was stronger than Matrine. Zhang et al. (121) synthesized glycyrrhetinic acid Matrine complex (**Figure 8E**) through esterification of 18α-glycyrrhetinic acid, 18β-glycyrrhetinic acid and 13-hydroxyethoxymatrine. The compound had better inhibitory effects than Matrine in MCF-7 and SMMC-7221 cells.

### 4.7 Evodiamine Derivatives

**Figure 9:**
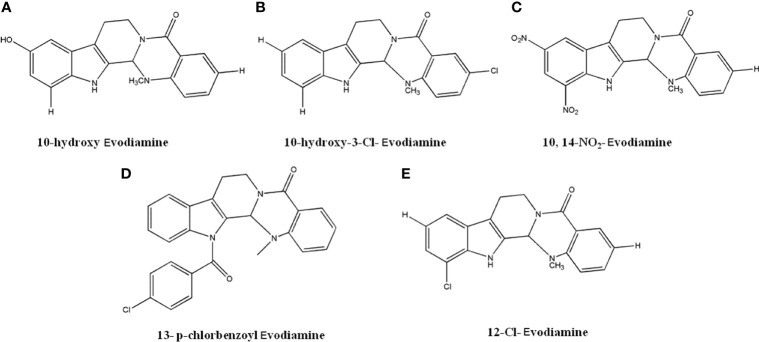
The structure of Evodiamine derivatives. **(A)** 10-hydroxy Evodiamine. **(B)** 10-hydroxy-3-Cl-Evodiamine. **(C)** 10,14-NO2-Evodiamine. **(D)** 13-p-chlorbenzoyl Evodiamine. **(E)** 12-Cl-Evodiamine.

Several novel derivatives of Evodiamine exhibited favorable anti-tumor activity. Dong et al. (122, 123) reported that compounds with hydroxy group substitution on the C-10 position of A ring (**Figure 9A**) and chloride group substitution on C-3 position of E rings (**Figure 9B**) had significant anti-cancer activity. The compounds showed better effects than Evodiamine in A549, MDA-MB-435, and HCT116 cells, with IC_50_ values lower than 0.003 µM. Studies also identified that compound A with simultaneous introduction of -NO_2_ from C-10 and C-12 on the A ring of Evodiamine (**Figure 9C**) and compound B with p-chlorobenzoyl group introduced to N-13 on ring B (**Figure 9D**) had significant anti-tumor effects. In MDA-MB-435 cells, the GI_50_ values were 0.16 µmol/L and 0.049 µmol/L, respectively. It was also suggested that compound with chloride substitution on C-12 on A ring (**Figure 9E**) had optimal inhibitory effects against MDA-MB-435 cells, with GI_50_ value less than 0.003 µM (122, 123).

### 4.8 Apigenin Derivatives

**Figure 10:**
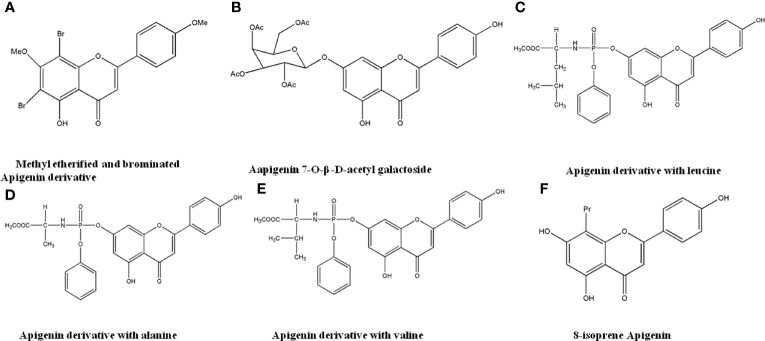
The structure of Apigenin derivatives. **(A)** Methyl etherified and brominated Apigenin derivative. **(B)** Apigenin 7-O-β-D-acetyl galactoside. **(C)** Apigenin derivative with leucine. **(D)** Apigenin derivative with alanine. **(E)** Apigenin derivative with valine. **(F)** 8-isoprene Apigenin.

Apigenin has good anti-tumor activity, but its water solubility and intestinal absorption are poor, which limits its therapeutic application. Therefore, it is structurally modified to improve its anti-tumor activity. Xiang et al. (124) performed structural modifications on Apigenin to generate 6 new Apigenin derivatives. The results suggested that methyl etherification and bromination derivatives (**Figure 10A**) have optimal water solubility and intestinal absorption, with 12.09-fold stronger anti-cancer effects than Apigenin. Chen et al. (125) synthesized 4 new Apigenin derivatives by reacting 4’-O-benzylapigenin with bromoacetylglucose and bromogalactose, respectively. The apigenin-7-O-β-D-acetylgalactoside (**Figure 10B**) has the most significant antiproliferation effects against MCF-7 and HL-60 cells. Han et al. (126) reported that derivatives with leucine, alanine and valine as substituents (**Figures 10C-E**) have potential anti-cancer effects to HeLa cells. Daskiewicz et al. (127) introduced isoprenyl group to the 5-position of the Apigenin molecule, and after molecular rearrangement, 8-prenyl Apigenin (**Figure 10F**) was obtained. The compound had significant anti-proliferative effects and induced apoptosis in HT-29 cells.

### 4.9 Artemisinin Derivatives

**Figure 11:**
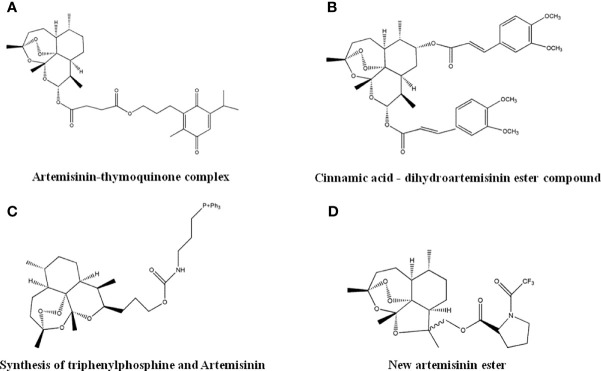
The structure of Artemisinin derivatives. **(A)** Artemisinin-thymoquinone complex. **(B)** Cinnamic acid-dihydroartemisinin ester compound. **(C)** Synthesis of triphenylphosphine and Artemisinin. **(D)** New artemisinin ester.

As an anti-malarial drug, Artemisinin exhibits anti-tumor activity, but its clinical application was limited by poor water solubility and bioavailability. Several Artemisinin derivatives, after structural optimization, showed enhanced anti-cancer effects. Researchers have combined the short-chain ubiquinone compounds Thymoquinone, with Artemisinin, and found that the resulting Artemisinin-Thymoquinone hybrid (**Figure 11A**) was effective against colorectal cancer. The inhibitory activity of HCT-116 and HT29 cells was better than 5-Fu treatment with less cytotoxicity (128). It was reported that the cinnamic acid-dihydroartemisinin ester hybrid (**Figure 11B**) had selective cytotoxic effects to lung cancer cells (129). Mitochondria play an important role in tumorigenesis and development. The synthetic product of triphenylphosphine and Artemisinin (**Figure 11C**) can introduce Artemisinin into mitochondria and enhance its anti-tumor activity (130). The new Artemisinin ester compounds synthesized by the reaction of acid chloride or acid anhydride (**Figure 11D**) have enhanced anti-tumor activity with good safety and thermal stability.

## 5 Concluding Remarks

A variety of natural products have shown favorable anti-tumor activity, providing new insights and core structures for anti-cancer new drug development. While natural products have low anti-cancer activity, poor water solubility, and poor absorption, structural optimization allows the development of lead compounds with high anti-cancer efficacy. The anti-cancer molecular mechanism of natural products and their derivatives are still not well understood, which hinders their clinical application. With the progress of in-depth research on natural products, more potential derivatives will be developed and will have broad application in cancer treatment.

## Author Contributions

H-LC designed the research study. MG, JJ, and DZ wrote the manuscript. ZR, X-YS, L-YJ, Y-DW, LH, and Y-HL collected the references. L-QC, Z-JH, LL, R-KM, Y-FL, and K-KS analyzed the data. A-HL and H-LC revised the paper. All authors contributed to editorial changes in the manuscript. All authors read and approved the final manuscript.

## Funding

This work was supported by the National Natural Science Foundation of China (U1932130); the Key Program of Shaanxi Provincial Science and Technology Department (2022ZDLSF05-15, 2021SF-303); the Key Program of Shaanxi Provincial Education Department (20JS134); the Program of Shaanxi Administration of Traditional Chinese Medicine (2019-ZZ-ZY009); the Key Program of Weiyang District Bureau of Science, Technology and Industry Information Technology (201928).

## Conflict of Interest

Author A-HL is employed by Shaanxi Pharmaceutical Holding Group Co., LTD, China. Author H-LC previously acted as an advisor for Shaanxi Pharmaceutical Holding Group Co., LTD, China.

The remaining authors declare that the research was conducted in the absence of any commercial or financial relationships that could be construed as a potential conflict of interest.

## Publisher’s Note

All claims expressed in this article are solely those of the authors and do not necessarily represent those of their affiliated organizations, or those of the publisher, the editors and the reviewers. Any product that may be evaluated in this article, or claim that may be made by its manufacturer, is not guaranteed or endorsed by the publisher.
